# Impact of Anti-CD20 therapies on the immune homeostasis of gastrointestinal mucosa and their relationship with *de novo* intestinal bowel disease in multiple sclerosis: a review

**DOI:** 10.3389/fphar.2023.1186016

**Published:** 2023-05-30

**Authors:** A. Quesada-Simó, A. Garrido-Marín, P. Nos, S. Gil-Perotín

**Affiliations:** ^1^ Department of Neurology, Hospital Universitario Dr. Peset, Valencia, Spain; ^2^ Gastroenterology Unit, Hospital Universitario y Politécnico La Fe, Valencia, Spain; ^3^ Department of Neurology, Hospital Universitario y Politécnico La Fe, Valencia, Spain

**Keywords:** ocrelizumab, rituximab, ulcerative colitis, crohn’s disease, microscopic colitis, anti-S1P

## Abstract

Multiple sclerosis (MS) and inflammatory bowel disease (IBD) are autoimmune disorders characterized by inflammatory episodes affecting the brain and the gastrointestinal (GI) tract, respectively. The frequent association between MS and IBD suggests that both conditions may share common pathogenic mechanisms. However, different responses to biological therapies indicate differences in immune mechanisms of inflammation. Anti-CD20 therapies are high efficacy treatments increasingly used to control inflammatory bursts in MS, but they may alter GI homeostasis and promote the development of bowel inflammation in susceptible individuals. This review analyzes the mechanistic association between immunity in MS and IBD, the effect of anti-CD20 therapies on the gut microenvironment, and provides recommendations for early detection and management of GI toxicities in the context of B-cell depletion in MS patients.

## Introduction

Multiple sclerosis (MS) is a highly prevalent chronic inflammatory disorder in the young population. It is characterised by an altered immune response against the central nervous system (CNS) that leads to demyelination and, to some extent, neurodegeneration, negatively affecting quality of life and functional independence. Inflammatory Bowel Diseases (IBD) are caused by an abnormal immune response to the gut microbiome, involving genetic and environmental factors. Ulcerative colitis (UC) and Crohn’s disease (CD) comprise the two main idiopathic conditions in this group. UC consists of diffuse inflammation of the colonic mucosa, it commonly affects the rectum and can extend proximally in a continuous pattern, affecting any part of the colon. CD is characterised by transmural inflammation and can affect the entire gastrointestinal (GI) tract, with the terminal ileum and colon being the most frequent locations. IBD can also associate with extraintestinal manifestations and increased risk of colorectal cancer. In this study, we review the existing literature on the association between MS and IBD, the common and distinct pathogenic mechanisms of both conditions, and the potential detrimental effect of anti-CD20 therapies on the inflammatory gut microenvironment. From all reported cases, we extract the information that might help recognizing anti-CD20-induced GI toxicity and optimizing the management of concurrent MS and IBD.

## Association between inflammatory bowel diseases and multiple sclerosis

The estimated prevalence of MS in patients with IBD is 0.7% and is approximately 0.3% in MS patients who develop IBD—with similar risk of developing CD or UC –, both slightly higher figures than the described in the general population (Wang et al., 2022). This association might be explained by an underlying common pathogenic mechanism.

**FIGURE 1:**
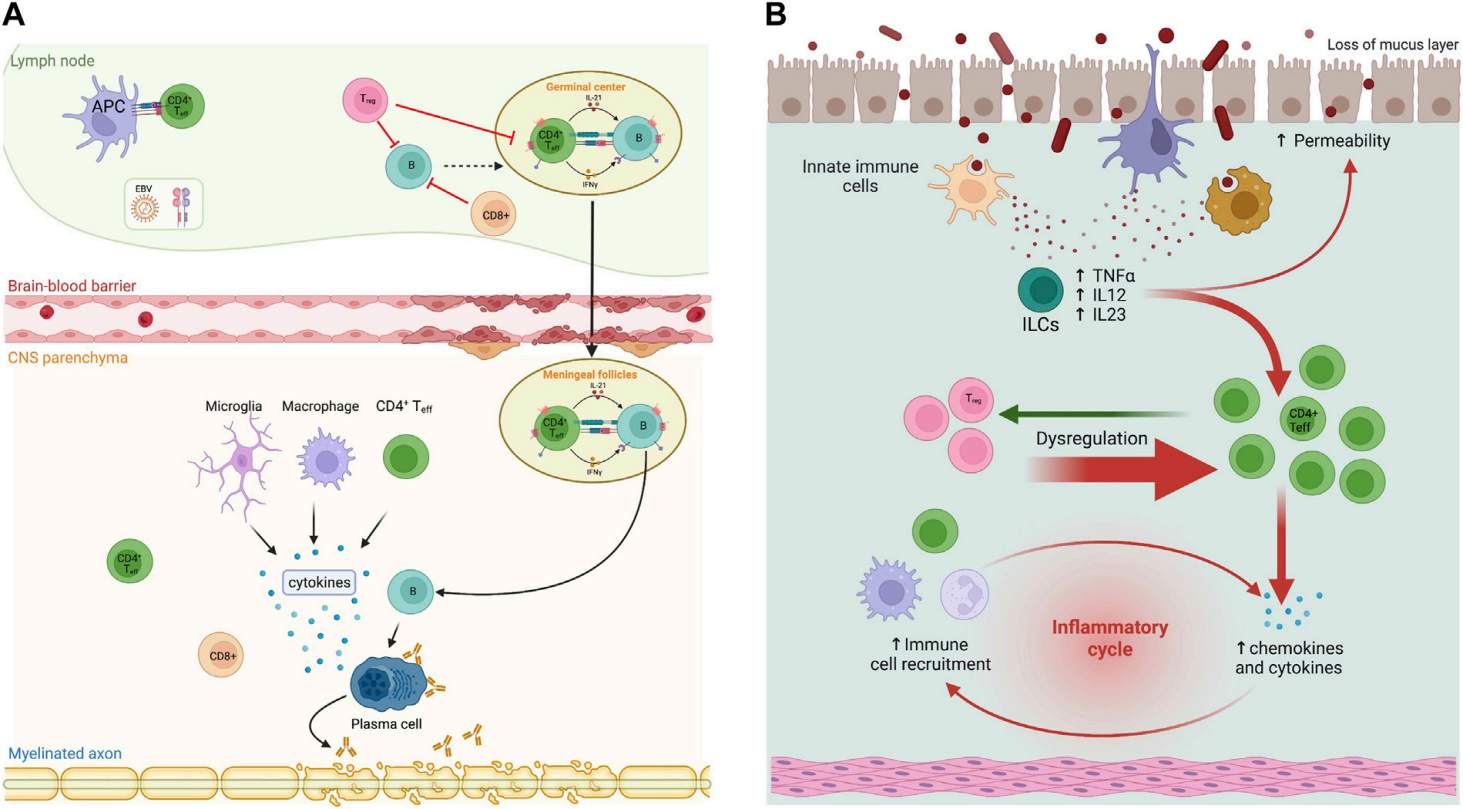
Immunopathogenesis in multiple sclerosis (MS) and inflammatory bowel diseases (IBD). **(A)** In MS patients, B and T cells interact in the periphery and central nervous system (CNS) to contribute to pathogenesis. In secondary lymphoid organs, EBV-infected B cells escape suppression by CD8^+^ and T regulatory (Treg) cells. Activated B cells in germinal centres interact with follicular T cells (Th) and differentiate into pathogenic memory B cells. These B cells activate Th effector cells (Th1 and Th17) that in turn infiltrate the CNS through the distinct expression of CXCR3, CCR6, and/or VLA4, as well as pro-inflammatory cytokines. In the CNS, T and B cells reunite in follicle-like structures resulting in clonal expansion. Plasma cells derived from B cell differentiation produce potential harmful antibodies. Cytotoxicity derived from CD8^+^ cells and clonal antibodies expand inflammation and produce demyelination. **(B)** Generally, Th17 and Treg cells modulate the proliferation of each other to maintain balance in GI mucosa. Th17 cells that release IL-17 are a strong pro-inflammatory factor, while Treg cells play a critical role in preserving immune homeostasis and establishing inflammation in response to foreign or non-pathogenic antigens such as commensal bacteria. Failure of Treg cell function can lead to IBD. A recently described cell type, innate lymphoid cells (ILCs) also provide host protective immunity in mucosal tissues, although mononuclear phagocytes have an important role in the activation of pathogenic ILCs. Modified and made with Biorender.

The pathophysiology of MS involves a complex relationship between different lymphocyte subpopulations and other immune cells, leading to an increased pro-inflammatory response against myelin antigens and other factors (Bar-Or and Li, 2021). B cells are thought to play a crucial role in the pathogenesis of MS by increasing the secretion of immunoglobulins directed against myelin antigens and acting as antigen-presenting cells to pathogenic T cells, thereby triggering a proinflammatory response (Weber and Hemmer, 2009; Weber et al., 2011) (Figure 1A). Similarly, the pathophysiology of IBD involves an alteration of the immune system, where innate lymphoid cells (ILC) are particularly relevant. These are phenotypically T cells but lack T-cell receptors and function by controlling the levels of the microbiota, regulating the repair of the intestinal mucosal barrier, and modulating the immune response. In IBD, there is upregulation of most pro-inflammatory subpopulations of ILC (Saez et al., 2021) (Figure 1B). Elevated levels of IL-17 in MS and IBD also suggest a common pathogenesis (Shahmohammadi et al., 2018).

Alteration of intestinal permeability has been observed in MS patients (Buscarinu et al., 2018) that presented with number of CD8+/CD161+ T cells in peripheral blood compared with healthy donors. These CD8+/CD161+ lymphocytes have also been associated with the mucosa-associated T lymphocyte (MALT) variant and are believed to be involved in the pathogenesis of IBD (Buscarinu et al., 2018).

MS and IBD may also share a genetic background that predisposes individuals to suffer both conditions. Four single nucleotide polymorphisms shared by MS and IBD have been described rs13428812, rs116555563, rs13428812, and rs9977672 in GWAS studies (Yang et al., 2021). Comparably, some environmental factors seem to increase the risk of MS and IBD such as vitamin D deficiency, smoking, high socioeconomic status, cold weather, or infections caused by microorganisms such as *Campylobacter jejuni* (Zois et al., 2010; de Felice et al., 2015).

Despite the potential similarities in the pathophysiology of MS and IBD, the disparity in the efficacy of immunosuppressive treatments suggests divergent underlying mechanisms (Rodrigues et al., 2010; de Felice et al., 2015; Camara-Lemarroy et al., 2018). From now on, we will focus on the potential effect of anti-CD20 therapies on GI mucosa and its relationship with IBD in MS, but also, in non-MS patients.

## Anti-CD20 monoclonal antibodies as therapies in MS and IBD

Anti-CD20 monoclonal antibodies are high effective treatments among the disease-modifying therapies (DMTs) available for MS. CD20 is a transmembrane protein expressed in B cells. Anti-CD20 monoclonal antibodies reduce the number of CD20^+^ B cells in peripheral blood and significantly decrease inflammation in MS (Margoni et al., 2022).

Rituximab (RTX) is a chimeric monoclonal antibody not approved by the Food and Drug Administration (FDA) or the European Medicines Agency (EMA) for its use in MS, however it is widely used off-label. A single course of RTX reduced brain inflammation and clinical relapses over a 48-week period compared to placebo in patients with relapsing MS (RMS) (Hauser and Cree, 2020). Compared to injectable DMTs or dimethyl fumarate (DMF), patients treated with RTX had lower rates of relapse and gadolinium-enhancing lesions (GELs) on magnetic resonance imaging (MRI) (Granqvist et al., 2018). Ocrelizumab (OCR), a humanized anti-CD20 monoclonal antibody, was approved by the FDA in 2017 and the EMA in 2018 for the treatment of active RMS and primary progressive MS (PPMS). OCR has been associated with lower disease activity (measured as annualized relapse rate-ARR) and slower progression compared to IFNβ-1a in patients with RMS (Hauser and Cree, 2020). It has also been associated with lower rates of clinical progression and MRI compared to placebo in patients with PPMS (Montalban et al., 2017). Ofatumumab (OFA) is a fully human anti-CD20 monoclonal antibody approved by the FDA in 2020 and the EMA in 2021 for active RMS. OFA has been associated with lower ARR in patients with RMS compared to oral DMT teriflunomide (TFM) (Hauser and Cree, 2020). Several meta-analyses have compared the efficacy and safety of the different anti-CD20 monoclonal antibodies available with that of non-biological therapies for RMS (Asha et al., 2021). Lower ARR was observed with all anti-CD20 therapies (RTX, OCR, OFA) compared to non-biological treatments. ARR was significantly lower with OCR therapy compared to IFN, TFM and placebo; with OFA compared to TFM, and with RTX compared to placebo. Only OCR was associated with lower rates of severe adverse events compared to other non-biological drugs. In terms of efficacy and safety, there were no differences between the three anti-CD20 drugs.

Among the available anti-CD20 inhibitors, only RTX has been evaluated for IBD treatment, specifically in UC. RTX had a non-significant effect on remission rates in patients with steroid-refractory UC. A short-term response to RTX was observed, but it was not sustained over time (Leiper et al., 2011).

## The effect of anti-CD20 therapies on the GI mucosa could predispose to *de novo* IBD

Anti-CD20 monoclonal antibodies can cause GI side effects with IBD-like symptoms and epithelial lesions in MS and non-MS patients. Evidence of this association comes from case reports suggesting that RTX and OCR treatment may not only cause IBD exacerbation, but also direct GI toxicity in the form of *de novo* UC and CD (Shahmohammadi et al., 2018; Lee et al., 2020; Barnes et al., 2021; Au et al., 2022; Sunjaya et al., 2020; Klug et al., 2022; el Fassi et al., 2008; Tsuzuki et al., 2021; Cavalcanti et al., 2020; Shankar et al., 2019; Barreiro Alonso et al., 2019; Morita et al., 2019; Uzzan et al., 2018; Varma et al., 2017; Fraser et al., 2016; Lipka et al., 2016; Bhalme et al., 2013; Sekkach et al., 2011; el Fassi et al., 2011; Vallet et al., 2011; Ardelean et al., 2010; Goetz et al., 2007; Blombery et al., 2011). However, in the reported cases many other environmental factors could be involved in the development of GI adverse effects, such as the simultaneous use of drugs or the presence of concomitant pathologies that could contribute to local tissue damage (D’Haens, 2007).

**FIGURE 2:**
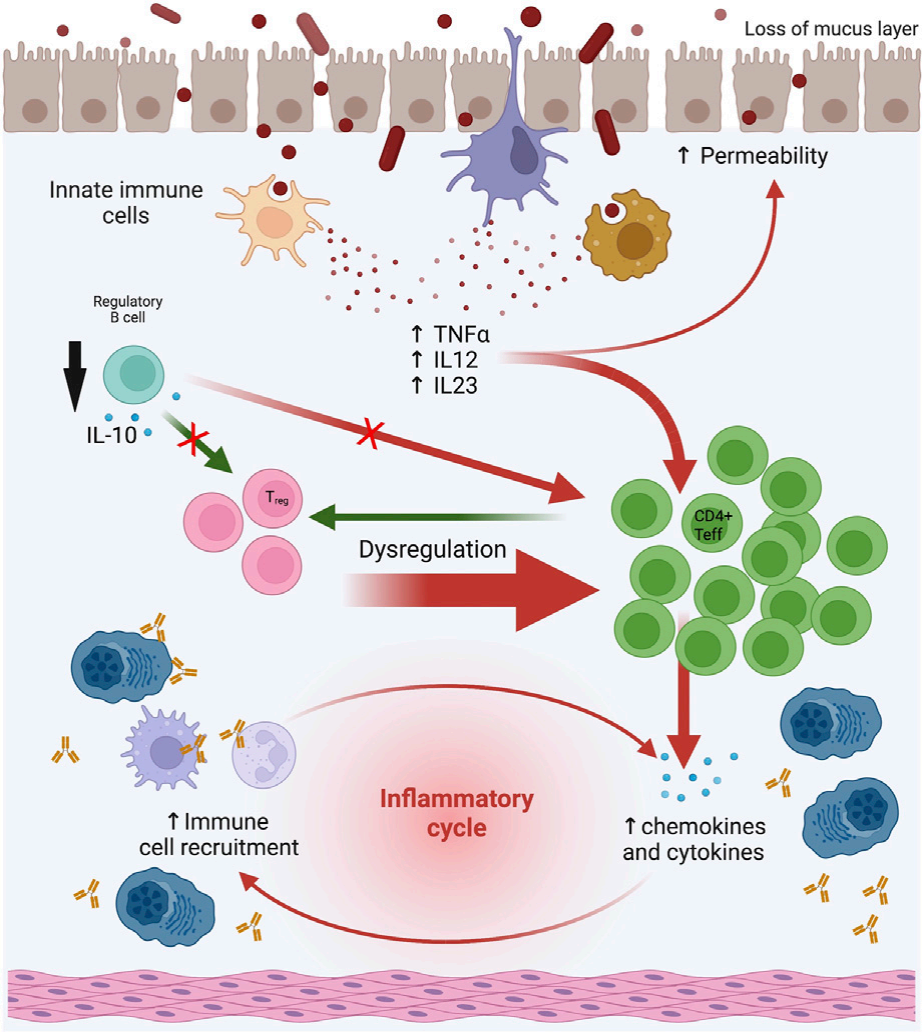
Effect of anti-CD20 therapies on GI mucosal microenvironment. The deleterious effect of anti-CD20 therapies could be explained by the depletion of the B-cell compartment because B cells in the GI mucosa may act as regulatory with a potential protective effect. B cells regulate cellular production of TNFα in colonic inflammation, produce anti-inflammatory cytokines such as IL-10, and have an adjuvant effect on regulatory T cell (Treg) function. Abbreviations: GI: gastrointestinal. Modified and made with Biorender.

To link anti-CD20 therapies with a deleterious GI effect, it should be demonstrated that these drugs have a direct effect on the GI tract. Relevant findings on the potential role of anti-CD20 therapies on IBD onset come from mouse models. Intestinal mucosal immunity relies on a balance between pro- and anti-inflammatory stimuli from the innate, humoral, and cell-mediated immune systems and, it has been shown that the presentation of antigens by B cells may be beneficial in IBD (Mizoguchi et al., 2000) (Figure 2). In a mouse model of MS with experimental autoimmune encephalomyelitis (EAE) the contribution of B cells in disease onset and progression has been shown (Matsushita et al., 2008), and regulatory B cell depletion in these animal models was linked to colitis (Barnes et al., 2021).

Treatment with RTX in control mice altered T cell populations in the mesenteric lymph nodes (Zhao et al., 2021). Specifically, there was an increase in Th1 (CD4+/IFNγ+) and Th17 (CD4+/IL-17A+) lymphocytes. Th1 cells promote the cellular inflammatory response by activating macrophages, NK cells, and B cells, while Th17 cells induce the expression of adhesion molecules and proinflammatory cytokines. In addition, an increase in activated proinflammatory macrophages (CD11b+ F4/80+) was also observed in the mesenteric lymph nodes and spleen. In RTX-treated mice there was a change in microbiota composition with a reduction in diversity and other qualitative changes such as a reduction in *Lactobacillus reuteri* compared to the control group (Zhao et al., 2021). In mice with a dominant negative mutation in the TGFβ-II receptor (a model of autoimmune cholangitis/primary biliary cirrhosis), depletion of B cells improved autoimmune cholangitis but exacerbated colitis. Furthermore, there was an increase in serum concentrations of TNFα and IL-6, and an increase in mononuclear cells expressing TNFα in the colon, suggesting that B cells may regulate TNFα production in colonic inflammation (Moritoki et al., 2009). Finally, the potential effect of B cell in depletion of regulatory T cells (Treg) has been suggested after the recovery of Tregs in mucosa with B-cell adoptive transfer techniques in mice (Wei et al., 2005).

Genetic susceptibility mechanisms in the development of IBD after treatment with anti-CD20 drugs have been studied in a mouse model with a deficiency in the alpha i2 subunit of the G protein, known as Galphai2. These mice showed a reduction in type 2 transitional cells and B-1a B cells in the marginal zone, an increase in mature follicular cells and B1b B cells, and the occurrence of IBD-like colitis. The authors suggest that disruptions in the processes involving B cell development regulated by Galphai2 may increase the risk of IBD (Dalwadi et al., 2003).

Studies in human individuals also suggest that B cells may have a protective role rather than pro-inflammatory in IBD by producing the anti-inflammatory cytokine IL-10 (Klug et al., 2022; del Sordo et al., 2022). Goetz et al. found a higher production of IL-10 by mononuclear cells in the colonic lamina propria of patients not treated with RTX compared to those treated with RTX (Goetz et al., 2007). Furthermore, B cells in intestinal lymphoid tissue produce anti-inflammatory IL-4 and TGF-β, promote the removal of apoptotic cells from the GI tract, and control the amount of circulating autoantigens that may contribute to organ-specific autoimmunity (del Sordo et al., 2022; Vallerskog et al., 2007). Depletion of B cells can also disrupt B-T cell interactions, leading to Treg dysfunction and activation of Th1 and Th17 cells (Xu et al., 2014). Ardelean et al. suggested that infectious agents such as torovirus could contribute to the onset of inflammatory IBD in a host with depleted B cells, what should prompt to test the presence of torovirus and other pathogenic viruses in biopsy tissue (Ardelean et al., 2010; D’Haens, 2007).

## Characterization of GI toxicity and IBD-like conditions developed in patients receiving anti-CD20 therapies

Among the reported cases of GI toxicity in patients treated with anti-CD20 antibodies, the most common agent associated with IBD-like conditions was RTX because is widely used for diverse pathologies. RTX was prescribed for MS, haematological malignancies (lymphomas), Graves’ disease, granulomatosis with polyangiitis, rheumatoid arthritis, systemic lupus erythematous (SLE), UC, and nephrotic syndrome. In MS cases there was only one case described with RTX and 4 cases with OCR (Tables 1, 2). In a population-based retrospective cohort study from Iceland, 651 patients treated with RTX (for all indications) from 2001 to 2018 were studied and 7 developed IBD (2 CD:1 ileal and 1 ileal + pancolitis, 3 UC, and 2 undetermined IBD), a higher incidence (202/100,000 cases) than expected compared to the general population with a hazard ratio of 6.6 for developing IBD (Kristjánsson et al., 2021).

**TABLE 1:** Cases reported with the use of anti-CD20 and IBD in MS patients.

Reference	Age (sex)	Anti-CD20	Time to first symptoms	Clinical features	Complementary tests	Histopathology	Treatment	Follow-up
Lee et al. (2020)	43 (F)	OCR	6 months	Diarrhoea and abdominal pain	CT: transverse and sigmoid colitis	Superficial mucosa ulceration, congestion, chronic inflammation and submucosal fibrosis	Hydrocortisone	Total colectomy due to lack of response
					End.: nodular mucosa with white-yellow plaques			
Barnes et al. (2021)	56 (F)	OCR	18 months	Bloody diarrhoea and odynophagia	End.: deep ulcerations in oesophagus with inflammation from proximal until transverse colon	Colon: patchy chronic inflammation, moderately active with cryptitis and cryptica abscess without granulomas. No CD20^+^ detected in biopsy.	OCR discontinuation	3 months: resolution of inflammation, biopsy with microscopic colitis
						Oesophagus: mucosal ulceration with no viral inclusions or granulomas	Hydrocortisone iv followed by oral prednisone with tappering dose	9 months: normal colonoscopy and biopsy
Au et al. (2022)	45 (F)	OCR	5 years	Diarrhoea and abdominal pain	End.: active ileitis	Mild patchy ileal inflammation	Vedolizumab and OCR	Recurrence at 5 months; azathioprine was added
					Entero-MRI: active ileitis			
Shahmohammadi et al. (2018)	31 (F)	RTX	Not available	Abdominal pain, fever, bloody diarrhoea	End.: rectal mucosa erythema, superficial ulcers and blood loss from sigmoid colon to cecum	Distrained colonic mucosa, irregularity of crypts, lymphoplasmacytic infiltrate in lamina propria without granulomas (UC)	RTX suspension	Complete clinical resolution
							Hydrocortisone iv and 5-ASA	
Sunjaya et al. (2020)	47 (M)	OCR	Several weeks (not specified)	Fever and bloody diarrhoea	CT: mural thickening in rectum	Non specifical severe chronical inflammation	Corticoids iv followed by oral prednisone lowering dose	Clinical recurrence at 4 weeks with similar lesions in endoscopy. Corticoids iv and hydrocortisone enemas were reiniciated without improvement. Finally, segmental sigmoidectomy
					End.: haemorrhagic proctosigmoiditis with ulceration			
-	38 (F)	RTX	4 months after last cycle	Bloody diarrhoea, fever and perianal disease	End.: deep and superficial ulcers from ascendent colon to proximal sigma and deep ulcers, mucous bridges and a fistulous hole in anal mucosa at 2–3 cm from pectinate line	Colonic ulcers with granulation tissue. The features were compatible with Crohn disease	Intravenous corticosteroids followed by oral prednisone lowering dose and cefuroxime for the anal abscesses	Favourable evolution with initial treatment
					Pelvic MRI: four abscesses adjacent to anal canal, two of them with intersphinteric fistulas and one of them in communication with the anal canal			Maintenance treatment was initiated due to perianal disease with ustekinumab
Malloy et al. (2022)	40 (F)	OCR	not available	Postprandrial abdominal cramps	End.: mild patchy colitis	Histological evidence of severe rectal-sparing pancolonic inflammation	Initially mesalazine (discontinued due to intolerance)	Absence of clinical recovery despite treatment
					Posterior End.: severe with rectal-sparing and pancolonic inflammation		Mercaptopurine	It was also refractory to metilprednisolone and ciclofosfamide, finally requiring subtotal colectomy

5ASA: 5-aminosalicylic acid or mesalazine, CT: computerized tomography; End.: endoscopy; F: female; iv: intravenous; M: male; MRI: magnetic resonance imaging; OCR: ocrelizumab; RTX: rituximab; UC: ulcerative colitis.

**TABLE 2:** Cases reported with the use of anti-CD20 and IBD in patients with non-MS conditions.

References	Age (sex)	Disease	Anti-CD20	Time to symptoms	Clinic	Complementary tests	Histopathology	Treatment	Follow-up
Klug et al. (2022)	27 (F)	LP, UC, SC	RTX	2 months	Fever, abdominal pain, bloody diarrhoea	CT-PET: pancolonic hypermetabolism	Not available	Low dose corticoids	Clinical improvement
						CT: *lead-pipe* image			
el Fassi et al. (2008)	45 (F)	GD	RTX	7 days	Bloody diarrhoea, fever and joint pain	End.: UC	Mucosal inflammation, irregular crypts and cryptic abscesses with no granulomas. Absence of CD20^+^ cells. Persisting plasma cells and T cells	Prednisolone enemas during 14 days followed by 5-ASA for maintenance	Clinical improvement
						HLAB27-pANCA+			Restoration of CD20^+^ cells levels on biopsy after 3 months of treatment
Tsuzuki et al. (2021)	65 (M)	Gastric maltoma	RTX	4 months	Watery and bloody diarrhoea	CT: longitudinal hypertropia from terminal ileum to rectum	Wide inflammation with diffuse circumferential erosions, epithelial atrophy, cryptitis and cryptical abscess without granulomas. Lymphocytic infiltration with atypical lymphocytes and intraepithelial lymphocytes (similar to MC). No CD20^+^ cells. Presence of CD3^+^T, CD79a^+^B and CD68^+^ cells	Oral prednisolone during 2 weeks followed by tappering dose	Clinical improvement with complete symptom resolution after 3 months of treatment. Endoscopic improvement after 5 months of treatment
						End.: hyperaemia end erosions in gastric antrum; patchy ulcers with diffuse inflammation in terminal ileum and colon			
Cavalcanti et al. (2020)	45 (M)	Gastric ADC and NHL	RTX	Not available	Watery and bloody diarrhoea and abdominal pain	PET: hypermetabolism in terminal ileum and mesenteric lymph nodes	Nonspecific active ileitis	RTX discontinuation	Clinical worsening after additional RTX cycles. Good response to RTX withdrawal, budesonide and 5-ASA.
						End.: erythema, aphthous erosions and inflammation in terminal ileum	After additional RTX cycles: chronic active inflammation, cryptical abscess, granulation tissue in lamina propria. Total depletion of CD20^+^ cells in ileal mucosa with increase of CD3^+^ T cellularity intraepithelial and in lamina propria with moderate excess of enlarged macrophages in lamina propria	Budesonide and 5-ASA followed by 5-ASA for maintenance	At week 10, asymptomatic with good endoscopic control. After 30 months with 5-ASA still in remission
Shankar et al. (2019)	58 (F)	Tonsillar FL	RTX	3 years	Bloody diarrhoea, abdominal pain, oral ulcers and nodous erythema	PET: hypermetabolism in terminal ileum and mesenteric lymph nodes	Nonspecific severely active ileitis with non-caseating granulomas	Oral prednisone and ustekinumab (induction and manteinance)	4 weeks: pain and nodous erythema resolution with CRP and ESR normalization
						End.: inflammation with extensive ulceration in terminal ileum			6 months: End.: no inflammation signs, nor fistulas nor stenosis. PET: without activity in terminal ileum
						After additional RTX cycles			
						PET: new activity in sigmoid colon			
						Entero-MRI: ileo-colic fistula			
Barreiro Alonso et al. (2019)	55 (M)	MCL	RTX	2 years	Watery diarrhoea	End.: erythematous colon mucosa from rectum to cecum with some oedema and exudate. Erythematous terminal ileum with isolated ulcers	Nonspecific active chronic colitis and nonspecific chronic ileitis with granulation tissue	Loperamide	Clinical improvement with 5-ASA
								5-ASA after cessation of RTX	
Morita et al. (2019)	15 (M)	RNS	RTX	2 years	Abdominal pain, watery diarrhoea, weight loss and oral aphtas	CT: mural circumferential thickening from ileocecal union to ascending colon	Chronic colitis with severe active inflammation without granulomas nor inclusion bodies nor caseous necrosis	Mouth wash with 5-ASA and fasting therapy with parenteral nutrition	Clinical improvement after 5-ASA and fasting therapy
						End.: multiple punched-out ulcers and cobblestone pattern in ascending colon with patchy erosions from transverse colon to rectum. In endoscopic video-capsule, multiple erosions in small intestine		Infliximab for maintenance without cessation of RTX	No recurrence of nephrotic syndrome or Crohn’s disease after maintenance with infliximab and RTX.
Uzzan et al. (2018)	25 (F)	DLBCL	RTX	6 months	Epigastric pain	End.: normal colonic mucosa	Absence of CD20^+^ cells in plasma and colon lamina propria with normal T cells and plasma cells. Significant increase in CD19^+^ cells population (almost exclusively CD38hiCD27+ -gut resident plasma cells-, mostly IgA+). Lower CD19-/CD19+ ratio than controls	-	-
Varma et al. (2017)	80 (F)	SCL	RTX	3 months	Diarrhoea and fever	PET: ileal hypermetabolism	Patchy active mucosa inflammation with ulceration and multiple small granulomas, some of them with multinucleated giant cells	Budesonide	
						End.: abnormal proliferative tissue and ulcers in ileocolic union, isolated ulcer in hepatic angle and left colon		Surgical removal of ileocolic union mass, observing small intestine involvement and fistulas	
						CT: inflammatory mass in right colon			
	74 (F)	NHL	RTX	2 years	Diarrhoea, abdominal pain, fever, weight loss and right iliac fossa pain	CT: terminal ileal mural thickening	Active ileitis with ulceration. Active focal mucosal inflammation with ulcer and granuloma in right colon	Budesonide	Recurrence of fever and abdominal pain after 3 weeks. Initiation of hydrocortisone iv and tappering dose of oral prednisone and methotrexate
						End.: ileal inflammation, lineal ulceration. Normal colon mucosa			
Fraser et al. (2016)	24 (F)	GPA	RTX	2 years	Perineal ulceration	End.: normal colon mucosa	Perineal skin: mixted inflammatory infiltrate	Infliximab and azathioprine	Fistula resolution
						MRI: acute inflammation	Colon: mild inflammatory changes with focal cryptitis with eosinophilic preponderance (no typical for IBD) and cryptical abscess		
						After new RTX cycle	After new RTX cycle: perineal skin: granulomas (CD)		
						MRI: wide fistulizing disease with recto-vaginal fistula			
Lipka et al. (2016)	62 (F)	MZL	RTX	Not available	Abdominal pain and diarrhoea	CT: diffuse colonic mural thickening with abdominal distension and areas of pneumatosis	Severe inflammation	Subtotal colectomy	Five years later, having received four RTX cycles due to lymphoma recurrence, proctectomy was needed because of clinical recurrence
Bhalme et al. (2013)	38 (F)	RA	RTX	11 weeks	Bloody diarrhoea	End.: moderate-severe colitis	Goblet cells depletion, active chronic inflammation and cryptical abscess. No CD20^+^ cells, low levels of CD19^+^ cells. Plasma cells and CD3^+^ CD138- T cells present	Corticoids and 5-ASA.	Lymphocytic restoration with biopsy and endoscopic normalization
								RTX discontinuation	
Sekkach et al. (2011)	34 (M)	B-SLE	RTX	3 weeks	Abdominal pain, nausea, watery diarrhoea	CT: intestinal mural thickening	Not conclusive for IBD.	RTX cessation	Complete resolution
						End.: erythematous-ulcerative pancolitis	CD20^+^ cell depletion in appendix biopsy		
el Fassi et al. (2011)	Age not available (F)	GD	RTX	18 months	Diarrhoea	End.: low grade colonic inflammation			Follow-up with normal barium studies
	Age not available (F)	GD	RTX	After 2nd infusion	Bloody diarrhoea	End.: UC in distal colon	-	5-ASA	Endoscopic normalization. Colonic B cell restoration after 170 days
						ANCA low titers			
Vallet et al. (2011)	66 (F)	RA	RTX	2 years	Mucous diarrhoea	CT: mesocolon inflammation	-	Ganciclovir for 7 days	Diarrhoea resolution after the first week of treatment.
						End.: superficial ulcerations		Valaciclovir for 14 days plus human Ig iv. infusion	
						CMV +			
						Plasma: Absence of B cells, normal T cells, IgG 2.77 g/L			
Ardelean et al. (2010)	4 (M)	RNS	RTX	6 weeks	Abdominal pain, weight loss, bloody diarrhoea, oral ulcers, intermittent fever	Abdominal echography: severe pancolitis with mural thickening	Focal areas of cryptitis and inflammatory infiltrate in lamina propria with lymphocytes, plasma cells and some eosinophils, without granulomas or giant cells. Absence of CD19^+^ and CD20+ cells. Activation of mature CD3^+^ T cells, cytotoxic CD8^+^ T cells and Treg FOXP3+ cells	Prednisone with posterior tappering dose	Recurrence of diarrhoea after cessation of prednisone. Restoration of prednisone during 2 months and addition of azathioprine. After 7 months, endoscopic resolution
						End.: grade IV severe inflammation with deep ulcerations from descending colon to rectum. Moderate grade II-III inflammation in ascending colon			After 11 months, CD19^+^ and CD20^+^ cells levels were restored
Goetz et al. (2007)	58 (M)	UC	RTX	Days	Bloody diarrhoea, weight loss and fever	End.: severe continuous colitis from anus to sigmoid colon, with spontaneous bleeding, ulcers, oedematous granular mucosa and loss of haustration. Low CD20^+^ cell levels in plasma.	Dense monocytic inflammatory infiltrate in mucosa with CD3^+^ T cells and complete depletion of CD20^+^ cells. In lamina propria mononuclear cells culture: absence of IL-10	RTX cessation 5-ASA, corticoids and ciprofloxacin	Partial recovery
Blombery et al. (2011)	67 (M)	FL	RTX	2 months	Fever, cough, dyspnoea, watery and bloody diarrhoea	CT: lung consolidation, pancolitis and ileitis with diffuse colonic mural thickening and pericolic stranding adjacent to cecum and ascending colon End.: severe confluent inflammation from anorectal union to proximal border at 30 cm in sigmoidoscopy	Complete loss of tubules and almost complete loss of superficial epithelium without pseudomembranes. Moderate mononuclear infiltrate in lamina propria with some eosinophils. No submucosal involvement. Absence of CD20^+^ cells with CD3^+^ cells normal or elevated and CD68^+^ cells (macrophages) elevated	Hydrocortisone iv	Clinical worsening requiring subtotal colectomy 2 weeks after the onset of the symptoms. Finally, the patient died 4 weeks after due to pneumonia

5-ASA: 5-aminosalicylic acid or mesalazine; ADC: adenocarcinoma; B-SLE: bullous systemic lupus erythematosus; CD: Crohn’s disease; CRP: C reactive protein; CT: computerized tomography; DLBCL: Diffuse large B-cell lymphoma; End.: endoscopy; ESR: erythrocyte sedimentation rate; FL: follicular lymphoma; GD: Graves’ disease; GPA: granulomatosis with polyangiitis; LP: lymphoproliferative disease; MC: microscopic colitis; MCL: mantle cell lymphoma; MZL: Marginal zone B-cell lymphomas; NHL: non-Hodgkin lymphoma; PET: positron emission tomography; RA: rheumatoid arthritis; RTX: rituximab; SC: sclerosing cholangitis; SCL: small cell lymphoma; RNS: refractory nephrotic syndrome; UC: ulcerative colitis.

**FIGURE 3:**
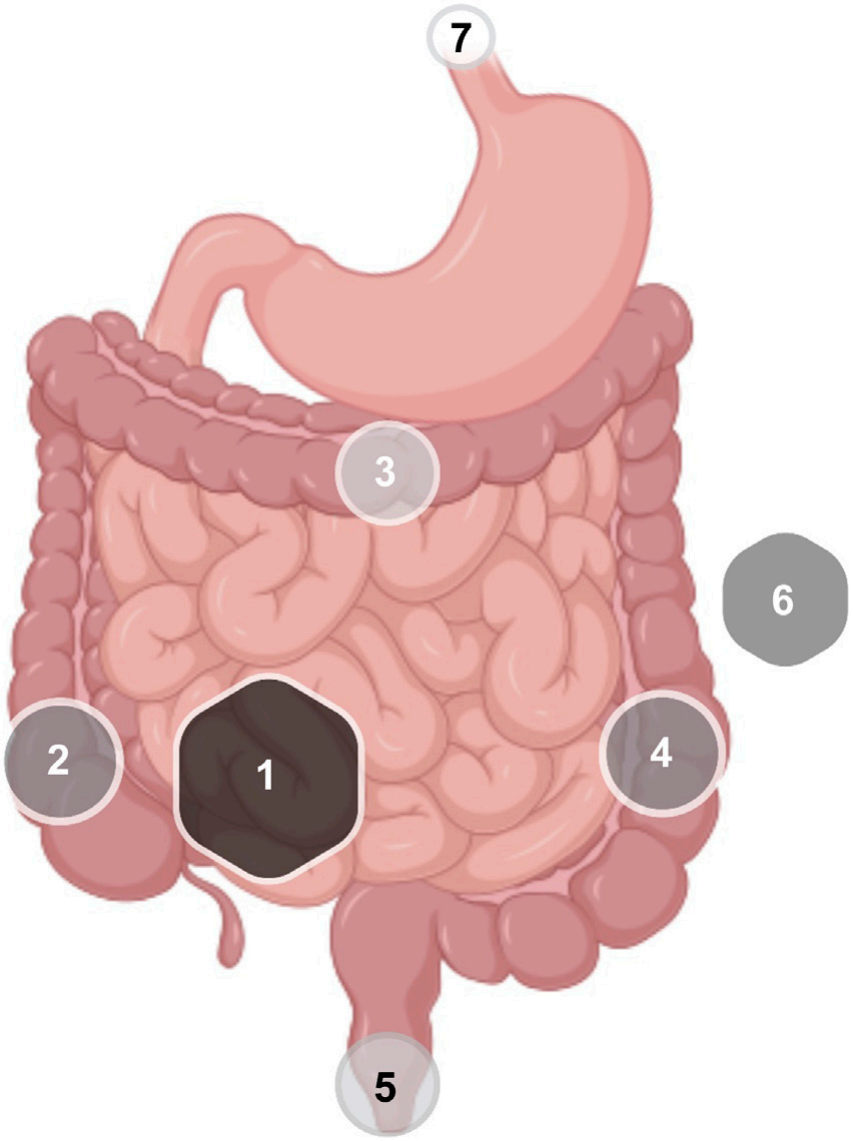
Frequency of distribution of GI inflammatory lesions in IBD associated with anti-CD20 therapies. Frequency was proportional to the diameter of the geometric figure: 1: Terminal ileum; 2: ascending colon; 3: transverse colon; 4: descending colon; 5: rectal-anal; 6: pancolitis; 7: esophagus (GI: gastrointestinal; IBD: intestinal bowel disease).

Summarizing all case reports, anti-CD20-induced enterocolitis was commonly characterized by nonspecific symptomatology, generally severe watery diarrhea (>20 stool/day) - that could be bloody -, abdominal pain, without concomitant involvement of other organs (Mallepally et al., 2019). Almost all patients with grade 3 diarrhea had macroscopical abnormalities on endoscopy. Regarding complementary tests, nonspecific markers of inflammation such as leukocytosis, elevated erythrocyte sedimentation rate (ESR) and C-reactive protein (CRP), as well as anemia, were frequent findings in the blood test. In a case series of colitis in patients treated with RTX (n = 93; 36 for inflammatory pathology and 35 for non-inflammatory pathology), DNA from cytomegalovirus (CMV) was found in two biopsy specimens (Kmeid et al., 2022). The most common locations of GI involvement were the proximal colon and the distal ileum, although it was not uncommon to demonstrate pancolitis (Figure 3).

In a third of patients in the case series described by Mallepally et al., , inflammation was restricted to the proximal part of the left colon, making evaluation by sigmoidoscopy insufficient and requiring a complete colonoscopy for diagnosis. Regarding the computerized tomography (CT) evaluation, only 57% had abnormal findings that demonstrated a poor correlation with symptoms and endoscopy. Therefore, the combination of CT and endoscopy together provided more information than using them separately (Mallepally et al., 2019).

Based on pathology findings in biopsy samples, some authors suggest that RTX could lead to ileocolitis through a variety of pathophysiological mechanisms related to deregulation of host immunity, in addition to a variety of environmental factors (Blombery et al., 2011). The most characteristic finding after treatment with anti-CD20 drugs was the depletion of CD20^+^ B cells in both the blood and the intestinal mucosa, which were reversible after discontinuation of the drug. Suppressed B cell levels after OCR infusion returned to pre-treatment levels with a median recovery time of 72 [27-175] weeks (Greenfield and Hauser, 2018). Similarly, serial study of biopsies after cessation of RTX resulted in the restoration of CD20^+^ B lymphocyte levels and the resolution of colitis (Zhou et al., 2018). Uzzan et al. observed the persistence in the lamina propria of short-lived CD19^+^ plasma cells despite the absence of CD20^+^ cells after 6 months of treatment, suggesting the existence of a small fraction of circulating CD20-/CD19+ plasmablasts that were resistant to RTX (Uzzan et al., 2018).

Other microscopic patterns found in patients treated with anti-CD20 therapies resembled those in UC, CD, or microscopic colitis (MC). Lymphocytic colitis injury induced pattern (LCIP) is a term that describes a clinical picture of watery diarrhea with normal endoscopy and abnormal biopsy findings characterized by an increase in intraepithelial lymphocytes (>20/100 epithelial cells) associated with damage to the epithelial surface and loss of goblet cells. This pattern has been described in RTX-induced colitis, but it is not specific and has also been observed in colitis induced by checkpoint inhibitors, proton pump inhibitors, SSRI, and NSAIDs (Kmeid et al., 2022). In a study of 21 patients treated with RTX who developed *de novo* colitis, 12 (57.1%) had clinical and histological findings consistent with IBD (7 CD, 5 UC) and 9 (42.9%) had MC (Eckmann et al., 2020). In the pathological analysis of the Iceland registry, chronic and acute inflammation was observed, with greater granulomatous inflammation and crypt abnormalities than in RTX-untreated patients (Kristjánsson et al., 2021).

In the cases described in the literature, IBD generally occurs after a latency period between the administration of the drug and the onset of symptoms. In a retrospective descriptive study of cancer patients who had received treatment with RTX, symptoms of colitis began with a mean time of 8 months after the initiation of treatment (Mallepally et al., 2019), in agreement with the study by Eckmann and colleagues (Eckmann et al., 2020). Symptoms resolved within a few months after withdrawal from the drug. A case in which IBD developed only one month after the start of treatment has been reported (del Sordo et al., 2022).

## Risk and protective factors of anti-CD20-induced colitis in MS

Risk factors for anti-CD20-induced colitis in MS are unclear (Lee et al., 2020). Genetic susceptibility is difficult to assess *a priori*, unless the patient reports IBD in first or second-degree relatives (Lee et al., 2020). The previous use of more than one DMT has also been shown to relate to *de novo* IBD (Lee et al., 2020). In the Icelandic registry that included MS and non-MS patients, the mean age at the time of diagnosis of IBD was higher than expected compared to the general population (55 years). The age-adjusted hazard ratio (HR) for developing IBD after treatment with RTX was 6.6, unaffected by the type of RTX indication or the total dose received. The additional use of other immunosuppressants also increased the HR to 7.4 ^51^. Regarding blood test parameters, the degree of lymphopenia and hypogammaglobulinemia, but not the neutrophil counts, correlated with the development of colitis (Mallepally et al., 2019). Albshesh et al. reported 25 patients with primary hypogammaglobulinemia and IBD-like features. In 20 of these patients hypogammaglobulinemia preceded the diagnosis of the GI condition, suggesting that decreased levels of IgG could be a risk factor for developing IBD. (Albshesh et al., 2021).

## Proposed criteria for the diagnosis of anti-CD20-induced colitis

Neurologists and gastroenterologists should be vigilant of MS and IBD comorbidity during clinical practice, and a potential contributing effect of anti-CD20 therapies. Based on all reported cases, we suggest a list of criteria that could help identify IBD as an adverse effect of anti-CD20 treatment (Table 3) although other causes should be ruled out and it should be considered an exclusion diagnosis. At least three major criteria and two minor criteria should be accomplished.

**TABLE 3:** Proposed diagnostic criteria for anti-CD20 -induced *de novo* IBD.

Proposed diagnostic criteria for anti-CD20 *de novo* IBD
*Major criteria*
Exposure to an anti-CD20 drug in the previous year
Compatible symptoms that may include: fever, abdominal pain, watery or muco-bloody diarrhea.
CD20^+^ cell depletion in GI biopsy
Lymphoplasmacytic infiltrate (CD3^+^ T cells and CD79^+^ plasma cells) in the lamina propria
Clinical/endoscopic recovery after drug withdrawal and CD20^+^ cell recovery in intestinal mucosa
*Minor criteria*
Elevation of acute inflamation biomarkers in laboratory tests (CPR, ESR, lymphocytosis)
Compatible endoscopic findings: mucosal erythema with edema and patchy ulcers/erosions with predominant involvement of the ileum and proximal colon. Tendency to spare stomach and duodenum
Chronic active inflammation with cryptitis, goblet cell reduction, and superficial ulcers with areas of spared mucosa
Good response to glucocorticoid therapy
*Absence of other possible aetiologies*
Normal neutrophil count
Absence of infectious causes: CMV, *Salmonella*, *Shigella*, *Campylobacter*, *E. coli*, *C. difficile*.
Other comorbidities that could justify the pathology
Other drugs/toxics that could justify the pathology

## Treatment of anti-CD20-induced colitis and *de novo* IBD: Recommendations for MS patients

There was a great variability in the disease course and the therapies administered in all reported cases, so extracting a one-size-fits-all recommendation is challenging and inaccurate. Likewise, it was not always possible to have the certainty of the association between GI symptoms and anti-CD20 therapies.

In general, withdrawal of anti-CD20 therapies is recommended until the patient’s clinical condition improves. In the study by Eckmann et al., 95% of patients discontinued RTX after the diagnosis of colitis, and the majority received colitis-specific treatment with improvement in 85.7% of the patients (Eckmann et al., 2020). In 83% of the cases reported by Mallepally et al., RTX was not discontinued, and colitis was controlled symptomatically with non-biological agents. The management of the acute phase of colitis typically consisted of fluid therapy, intravenous hydrocortisone with subsequent change to oral prednisone in a decreasing regimen, and oral mesalazine (5-ASA) (Tsuzuki et al., 2021). Corticosteroid enemas were added in case of rectal or distal sigmoid colon involvement. If symptomatic treatment was not sufficient, immunosuppressants such as azathioprine or biologic therapies were used. Some cases were severe and refractory to medical treatment and required surgical treatment (Blombery et al., 2011; Mallepally et al., 2019; Lee et al., 2020).

In people with MS who develop colitis, withdrawal of anti-CD20 antibodies may increase the risk of inflammatory flares, and therefore, after a reasonable wash-out period and normalization of blood counts (total lymphocytes and, specifically, B-cell repopulation), DMTs should be reinitiated. If sustained treatment for IBD was required, it is recommended to use effective agents for both conditions noting that some agents that are effective for one disease may be harmful for the other, such as anti-TNFα therapies that can worsen MS or, alternatively, β−IFN that can exacerbate IBD. In the case reported by Barnes et al., there was a multidisciplinary discussion on the use of additional biological agents to treat IBD in MS patients. The use of vedolizumab was considered as well as the use of ustekinumab (Barnes et al., 2021).

Vedolizumab (VDZ) is a humanized monoclonal antibody that binds to the α4β7 integrin and blocks the trafficking of intestinal lymphocytes. It is effective for inducing and maintaining response and remission in patients with IBD compared to placebo (Feagan et al., 2013; Sandborn et al., 2013) in moderately-severely active UC and CD that have inadequate response, have not responded, or have not tolerated conventional treatment or anti-TNFα agents. The combination of VDZ with biological agents such as OCR has been reported, being safe (Barnes et al., 2021; Kristjánsson et al., 2021). However the safety information and indications for VDZ do not recommend it. It should be noted that VDZ may not be as effective as other drugs in addressing extraintestinal manifestations of IBD, such as erythema nodosum or episcleritis. Additionally, although to date there is only one case described in a HIV patient, VDZ requires closely monitoring for the potential appearance of progressive multifocal leukoencephalopathy (PML). Similarly, Natalizumab (NTZ), approved for the treatment of MS and CD patients (the latter only by the FDA and not the EMA), is also an α4-integrin inhibitor that targets both α4β1 and α4β7 integrins and prevents the entry of activated leukocytes both into the CNS and intestinal mucosa, thus it could be a potential therapy with a dual effect (Pagnini et al., 2017). However, its use in IBD is not extended due to the risk of PML, that in the case of NTZ is higher than with VDZ. In people with MS in which anti-CD20 therapies have to be discontinued but presented recent CNS inflammatory activity or risk factors for an aggressive course of disease, switching to NTZ after a negative serology for John Cunningham virus (JCV) seems reasonable.

Ustekinumab (UST) is an anti-IL-12/23 agent used to treat IBD. IL-12 and IL-23 contribute to the pathogenesis of MS in the early stages by promoting T cell differentiation and its entry into the CNS. Due to its mechanism of action, its effectiveness in MS was investigated in a placebo-controlled, double-blinded trial (Segal et al., 2008). However, UST did not impede radiologic activity or reduced mean EDSS values compared to placebo. The potential explanation relies on the fact that neutralizing these cytokines years after the disease onset, when Th1 and Th17 differentiation and migration to the CNS has already occurred, might be ineffective (Longbrake and Racke, 2009).

An interesting alternative to treat both MS and IBD effectively are the sphingosine-1-phosphate (S1P) modulators. S1P is a lipid that interacts with membrane receptors, the S1P receptors 1 to 5 (S1PR1-5). S1P plasma levels are elevated in lymphatic nodes and relatively low in other tissues and interstitial fluid, facilitating a gradient for the lymphocyte mobilization from the lymphatic nodes to the general circulation. However, S1P has more biological functions depending on the receptor subtype with which it interacts. For example, S1P takes part in lymphocyte migration, endothelial permeability, angiogenesis, apoptosis, and differentiation signalling, among others (Danese et al., 2018). In MS, available S1P receptor modulators are fingolimod (FGM), siponimod (SPM) (Mayzent^®^), ozanimod (OZA) (S1P1 and S1P5 selective) (Zeposia^®^), and ponesimod (PNE) (S1P1 selective) (Ponvory^®^). These agents have shown their effectiveness in MS in different clinical trials compared to placebo, IFNγ or TFM (Calabresi et al., 2014; Kappos et al., 2018; Comi et al., 2019; Kappos et al., 2021). Although there are preclinical and clinical studies using S1P modulators in IBD (Danese et al., 2018; Ladrón Abia et al., 2021), FGM, PNE or SPM have not been investigated in clinical trials (Longbrake and Racke, 2009).

OZA is the first S1P-receptor modulator approved for the treatment of UC. In the TOUCHSTONE trial, a phase 2 placebo-controlled trial, it was observed that more clinical remission occurred in UC patients treated with OZA and there was a higher mucosal healing ratio (Sandborn et al., 2021a). In the TRUE NORTH trial, a multicenter, randomized, double-blind, placebo-controlled, Phase 3 study, clinical remission and response were significantly higher among patients treated with OZA in both the induction and maintenance phases. (Sandborn et al., 2021b). In the STEPSTONE trial, a 12-week multicenter, open-label, uncontrolled, single-arm Phase 2 study with endoscopic, histological, and clinical improvements were observed in patients with moderate to severely active CD initiating OZA therapy. Phase 3 placebo-controlled trials in CD have been initiated (Feagan et al., 2020). The efficacy of other MS therapies to effectively control IBD has not been evaluated.

Thus, the authors’ recommendation, to be taken cautiously and discussed for each individual case, would be to remove anti-CD20 therapy if the proposed criteria in Table 3 are met. After a safe wash out period of more than 6 months or after B-cell repopulation, we suggest treatment with NTZ in the case of previous high inflammatory activity and confirmed absence of anti-JCV antibody titers, or with OZA in cases of previous moderate activity or at least 5 years of stable disease under anti-CD20 therapies. Combination therapies with specific MS and IBD drugs could be used, but due to the lack of information to date, the authors do not recommend that approach.

## Conclusion

Anti-CD20 monoclonal antibodies alter the immune environment of the GI mucosa with various degrees of inflammation and damage and could associate with *de novo* IBD in susceptible patients with diverse underlying diseases, including MS. The decrease in B cells, thought to maintain the immune homeostasis of the intestinal mucosa, could underlie this adverse effect. It is important to recognize this condition to discontinue the causative drug and, in the case of people with MS, substitute it by an effective therapy that could play a dual role in brain and gut.
